# MEG Signatures of a Perceived Match or Mismatch between Individual and Group Opinions

**DOI:** 10.3389/fnins.2017.00010

**Published:** 2017-01-23

**Authors:** Ivan Zubarev, Vasily Klucharev, Alexei Ossadtchi, Victoria Moiseeva, Anna Shestakova

**Affiliations:** ^1^Centre for Cognition and Decision Making, National Research University Higher School of EconomicsMoscow, Russia; ^2^Department of Neuroscience and Biomedical Engineering, Aalto UniversityEspoo, Finland; ^3^School of Psychology, National Research University Higher School of EconomicsMoscow, Russia

**Keywords:** MEG, FRN, social conformity, posterior cingulate cortex (PCC), vmPFC, anterior cingulate cortex (ACC)

## Abstract

Humans often adjust their opinions to the perceived opinions of others. Neural responses to a perceived match or mismatch between individual and group opinions have been investigated previously, but some findings are inconsistent. In this study, we used magnetoencephalographic source imaging to investigate further neural responses to the perceived opinions of others. We found that group opinions mismatching with individual opinions evoked responses in the anterior and posterior medial prefrontal cortices, as well as in the temporoparietal junction and ventromedial prefrontal cortex in the 220–320 and 380–530 ms time windows. Evoked responses were accompanied by an increase in the power of theta oscillations (4–8 Hz) over a number of frontal cortical sites. Group opinions matching with individual opinions evoked an increase in amplitude of beta oscillations (13–30 Hz) in the anterior cingulate and ventral medial prefrontal cortices. Based on these results, we argue that distinct valuation and performance-monitoring neural circuits in the medial cortices of the brain may monitor compliance of individual behavior to the perceived group norms.

## Introduction

Humans typically adjust their behavior to match the group norms. A number of functional magnetic resonance imaging (fMRI) studies demonstrated that being exposed to a group opinion conflicting with one's own opinion triggers activity in the medial prefrontal cortex (MPFC) and ventral striatum (Klucharev et al., 2009; Berns et al., 2010; Campbell-Meiklejohn et al., 2010; Izuma and Adolphs, 2013). Interestingly, the posterior MPFC has been also implicated in the generation of a so-called “reward prediction error” signal when the outcome of an action differs from the one that is expected (Holroyd and Coles, 2002; Nieuwenhuis et al., 2004; Cohen and Ranganath, 2007; Rushworth et al., 2007). This signal presumably guides the selection of future actions by updating expectations about action values. These findings suggest social conformity may be based on general action-monitoring and reinforcement-learning mechanisms (Klucharev et al., 2009, 2011; Shestakova et al., 2013).

Two electroencephalographic (EEG) studies (Kim et al., 2012; Shestakova et al., 2013) demonstrated that a mismatch between an individual opinion and the opinion of a group elicits feedback-related negativity (FRN), a frontally distributed negative polarity event-related brain potential (ERP) component associated with outcome evaluation and behavioral adaptation (see Walsh and Anderson, 2012, for a review). FRN amplitude is greater whenever the outcome of an action is worse than expected. It was argued thus that similar to other negative outcomes, the perceived mismatch between the individual and group opinions may activate the generic outcome-evaluation mechanism in the MPFC (Shestakova et al., 2013). Evidence supporting this hypothesis comes from the fact that the evoked response to an opinion discrepancy highly resembled FRN in terms of latency and scalp topographies. Furthermore, earlier fMRI studies showed a BOLD signal increase over the MPFC to perceived mismatch between the individual and group opinions that was highly similar to brain activations following negative outcomes in non-social tasks (Klucharev et al., 2009).

The neural source of the FRN itself, however, remains debated. While fMRI studies report a greater increase in the BOLD signal over the MPFC following negative outcomes, recent magnetoencephalographic (MEG) and EEG findings contested the MPFC origin of the FRN (Doñamayor et al., 2011, 2012b) and the closely related error-related negativity (ERN) (Agam et al., 2011), suggesting the more posterior source in the posterior cingulate cortex (PCC). Importantly, a combined MEG–EEG analysis localized error-monitoring activity at a more posterior medial source—in the PCC—in stark contrast to data obtained via fMRI on the same subjects (Agam et al., 2011). Thus, in the case of the FRN, the findings from time-resolved (EEG, MEG) and spatially precise (fMRI) neuroimaging methods cannot be integrated in a straightforward manner. In the current study, we used MEG source imaging to investigate the spatio-temporal dynamics of neural responses in the medial cortices (posterior MPFC vs. PCC) to cues indicating either match or mismatch between individual and group opinions.

A number of studies have linked the FRN to the increase in the power of ongoing theta-band (4–8 Hz) oscillations in the MPFC and several other frontal sites (Cohen et al., 2007; Cavanagh et al., 2010, 2012; van de Vijver et al., 2011; Narayanan et al., 2013). Furthermore, the FRN has been shown to reflect a degree of theta phase consistency and power enhancement over the MPFC (e.g., Cohen et al., 2007). It has also been demonstrated that an increase of the BOLD signal in the posterior MPFC may in fact correspond to perturbations in non-phase-locked oscillatory theta-band activity (Winterer et al., 2007; Meltzer et al., 2009).

Moreover, beta (13–30 Hz) band activity was associated with the reward feedbacks in a number of EEG (Cohen et al., 2007; Mas-Herrero et al., 2015) and MEG studies (Doñamayor et al., 2011, 2012b), suggesting that beta oscillations can selectively track salient and novel positive events in the environment. Therefore, we also analyzed oscillatory activity during matches between individual and group opinions to clarify further the role of beta band activity in the processing of positive social information, such as an agreement of opinions.

We hypothesized that (i) the perceived mismatch between individual and group opinions triggers the neural signatures of processing negative outcomes: evoked responses in the medial cortices (posterior MPFC or PCC) similar to the FRN (and an increase in the power of theta oscillations in the medial cortices). We also hypothesized that (ii) coherence between individual and group opinions elicits the neural signatures of processing positive outcomes: an increase in beta oscillations.

To test these hypotheses, we used a paradigm in which participants' initial judgments about the trustworthiness of faces were open to the social influence of a group opinion (Campbell-Meiklejohn et al., 2012). Participants rated the trustworthiness of faces, and after each rating, they were informed of the “average group rating” assigned to the face by a large group of people. This procedure allowed us to create the situation where participants' opinions either repeatedly matched or mismatched with the opinion of the group. To clarify neural signature of opinion discrepancy we compared MEG activity in trials in which the group rating differed from the participant's rating (*conflict* trials) with MEG activity in trials in which the group rating matched the participant's rating (*no-conflict* trials).

## Materials and methods

### Participants

Twenty female volunteers took part in the experiment (mean age 24.2; range 18–28; right-handed; with normal or corrected eyesight). All of the participants reported no history of neurological or psychiatric disease, drug abuse, or head trauma. The data of one participant was discarded from the group analysis due to a large number of artifacts. For participating in the experiment, the subjects received monetary compensation (the equivalent of 16 US dollars) which typically covered a day's food expenses for a single person in Moscow. The study gained approval from the research ethics committee of the St.-Petersburg State University. All participants were familiarized with the experimental procedure and signed the informed consent form.

We tested the participants' personality traits using the Eysenck Personality Inventory (Eysenck and Eysenck, 1994), the Sensation Seeking Scale (Aluja et al., 2010), a short version of the Big Five questionnaire (Gosling et al., 2003), the Mehrabian Conformity Scale (Mehrabian, 1997), individual levels of anxiety (Hajcak et al., 2003; Gu et al., 2010), the Locus of Control questionnaire (Rotter, 1966), and Spielberger's State-Trait Anxiety Inventory (Spielberger et al., 1970). We did not find any significant correlations between the behavioral results and the personality traits identified using the above tests and guidelines (*p* > 0.2).

### Stimuli and procedure

In the present study, we used a modified face judgment task (Campbell-Meiklejohn et al., 2012) where participants were instructed to rate the trustworthiness of faces. During MEG recording (session 1), each participant was presented with a series of 222 photographs of emotionally neutral female faces (face presentation = 2 s; inter-trial interval = 2.5–3.0 s; overall session duration = 35 min). During MEG recording (session 1), each participant was presented with a series of 222 photographs of emotionally neutral female faces (face presentation = 2 s; inter-trial interval = 2.5–3.0 s; overall session duration = 35 min). The stimuli comprised 222 digital photos of Caucasian female faces (age 18–35 years) taken in highly similar photographic style. The stimuli were taken from free Internet sources. The same set of stimuli was used previously in Klucharev et al. (2009) and Shestakova et al. (2013).

**Figure 1:**
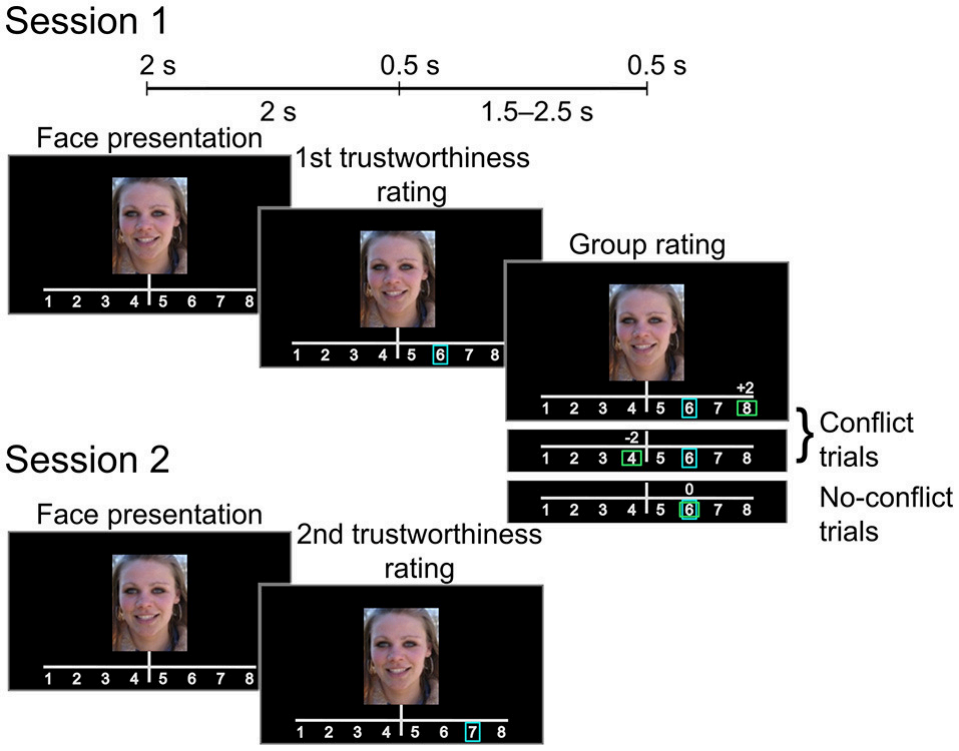
**Experimental design**. After giving the initial trustworthiness rating the subject was presented with either matching or mismatching group rating (Session 1). The subject rated the same set of faces again during the subsequent session (Session 2).

Each trial (see Figure 1) began with a 2-s presentation of a photograph of a female face (the face occupying approximately 60% of the image. Participants were instructed to decide whether to entrust the person viewed onscreen with a substantial sum of money (the equivalent of 1500 US dollars). They rated each face using an eight-point scale (1: very untrustworthy; 8: very trustworthy), indicating choice via the press of a numbered button. Each participant's rating (*initial rating*) was indicated on the screen by a blue rectangular frame immediately after the button press. Following this, the participant was informed how a large group of students from the same Russian university (*group rating*) rated the face. Similar to the initial rating, *group rating* was indicated by a green rectangular frame. In addition, the difference between the participant and the group rating values was displayed by a score shown above the scale (0, ±2, or ±3 points). Rectangles indicating both initial and group ratings appeared on the screen for 0.5 s. The group rating was displayed 2 s after the initial rating was made. If participant did not respond within 2 s after the face presentation, the trial ended and the text “Too late” appeared on the screen. Actual *group ratings* were generated pseudorandomly as R_g_ = R_0_ + M, where R_g_ was the *group rating*, R_0_ was the *initial rating* given by the participant, and M was a (pseudo) random modifier.

Our sampling scheme used an adaptive algorithm, ensuring that for 33% of the trials, the *group rating* agreed with the participants' *initial ratings* (no-conflict trials, M = 0), whereas in 67% of the trials, the *group rating* was above or below the participants' *initial ratings* by 2 or 3 points [conflict trials, M = (2, 3, −2, −3)]. Thus, the relative number of “more negative,” “more positive,” or “equal” *group ratings* was approximately the same for every participant. Participants were unaware of the real purpose of the experiment and were not informed about the mechanism for generating the group ratings. After the first MEG session, the participants took a 30-min break outside the testing area. Next, they were instructed to rate the same set of faces again (*subsequent rating*, session 2). Participants were not informed of why they were rating the same faces again, nor was it mentioned that the stimuli presented was the same.

Two 10-min blocks of resting-state activity was also recorded from participants before and after the first experimental session in order to estimate the task-independent brain-noise covariance matrix.

Three months after the MEG experiment, we asked the participants to rate the trustworthiness of the same faces again [subsequent session 3 data was collected for 15 of the 20 participants for another project (in preparation)].

The participants were debriefed about the study immediately after sessions 2 and 3. No subjects reported that the study was about social influence. None of the participants reported disbelief in the cover story. Subjects reported remembering 10–20 faces (14 subjects) or less from session 1, but they were unable to recall their own initial ratings.

### MEG acquisition and preprocessing

MEG data was recorded and processed in accordance with recent good practice guidelines for conducting MEG studies (Gross et al., 2013). We used a 306-channel Elekta Neuromag System comprising 102 magnetometers and 204 planar gradiometers, with a sampling rate of 1000 Hz. A low-pass filter with a 333 Hz cut-off frequency was applied to the data. To control for cardiac and eye-movement related artifacts, electrocardiographic (ECG) and electrooculographic (EOG) electrodes were mounted prior to MEG acquisition. Head movements were controlled using the continuous head position identification (cHPI) system. ECG electrodes were placed on the breastbone and on the axillary furrow approximate to the fifth rib. Vertical EOG (vEOG) electrodes were placed above and below the center of the left eye, and horizontal EOG (hEOG) electrodes were placed on the frontal processes of the left and right zygomatic bones. The ECG and EOG recordings were used as an additional source of information to project out artifacts.

Anatomical landmarks (NAS, LPA, RPA), cHPI coil positions, and 100 (±5) additional head shape points were digitized using the Polhemus Isotrak digital tracker system (Polhemus, Colchester, VT, USA). Participants were instructed to avoid movement and blink as little as possible during the experiment. Stimuli were presented on a semi-transparent display via a projector located outside the room. The distance between each participant's head and the display was 1.5 m. To ensure an equal distance between the frontal, the occipital sensors, and the participants' heads, a special cushion was used when necessary. The MEG was preprocessed using the Neuromag Maxfilter software by means of the temporally extended signal space separation algorithm (tSSS; Taulu and Hari, 2009), based on a temporal autocorrelation threshold of 0.9 and a segment length of 1 s. MEG data was then recalculated to compensate for head movements and to correspond to the default head origin coordinates of 0, 0, and 45 mm.

### Structural MRI acquisition and forward model

Individual structural MRI T1 images were collected for each participant using the 1.5-T Philips Intera scanner. Reconstruction of the cortical surfaces was performed using the FreeSurfer image analysis suite (http://surfer.nmr.mgh.harvard.edu). The resulting cortical surface meshes were imported into Brainstorm (Tadel et al., 2011) and down-sampled to 8000 vertices for further processing. Forward modeling was performed using the overlapping spheres method (Huang et al., 1999) as implemented in the Brainstorm software. Due to the unavailability of individual structural MRIs, the default MNI anatomy with 1 mm resolution was used for two participants.

### Analysis of behavioral data

To detect whether conflict with the group opinion led to changes in ratings during session 2, we grouped trials according to the direction in which the group rating differed from the initial rating (more positive, more negative, or matched the initial rating) and calculated the mean change between sessions for each of the groups. These scores were analyzed via a two-way ANOVA using within-subject factors of *conflict direction* (group's rating is more positive versus group's rating is more negative) and *conflict size* (smaller conflict ±2 points versus larger conflict ±3 points). We repeated the same analysis using a subset of faces with intermediate initial ratings (4 and 5) to account for possible artificial correlations caused by repeated measurements and the distribution of initial ratings.

### MEG data analysis

Analysis of the MEG data was performed using the Brainstorm package (Tadel et al., 2011) and custom-written Matlab routines (The MathWorks, Inc.). Prior to analysis, the recordings were down-sampled to a 500 Hz sampling rate. Event-related magnetic fields (ERF) and time-frequency maps were locked onto the presentation of the group rating. We grouped all epochs into *conflict* trials (i.e., when the participant's ratings did not match the group rating) and compared them to *no-conflict* trials (i.e., when the participant's ratings matched the group rating).

#### Sensor space event-related field (ERF) analysis

For the ERF analysis, we extracted epochs in the −200–800 ms time window. The direct current (DC) offset was removed for each trial by applying a zero-order polynomial detrend based on the pre-stimulus interval (−200–0 ms). To identify time windows for the relevant components of the evoked response that account for differences in activation between *conflict* and *no-conflict* trials, we computed a spatio-temporal cluster-based permutation test on the event-related field data separately for all magnetometers and all gradiometers. Cluster *p*-values were calculated as a probability of observing a cluster of equal or higher mass (positive and negative separately) over 10,000 random permutations. We used the time-window information of the resulting clusters to constrain the source analysis.

#### Source space ERF analysis

To localize the sources of the evoked responses, we used the Brainstorm implementation of the depth-weighted minimum-norm estimate algorithm (MNE; Hämäläinen and Ilmoniemi, 1994), loosely constrained to the individual cortical surface with penalization parameter of 0.2. For the group analysis, individual source-space ERF data were projected on the default MNI brain with a 1-mm resolution using the iterative closest point search algorithm as implemented in the Brainstorm software (Tadel et al., 2011). For each of the 8000 vertices, normalized source activations were obtained by computing the norm of three dipole moments in each direction and standardizing those values to pre-stimulus intervals of 200 ms (subtracting the mean and dividing by standard deviation of the baseline). For the purposes of group statistical analysis in the source space, the activity of each vertex over the time-windows was averaged where significant activations were observed in the sensor space. We also applied spatial smoothing using a 10-mm full-width half-maximum (FWHM) Gaussian kernel.

The resulting maps of source activations for *conflict* and *no-conflict* trials were submitted to a two-sample spatio-temporal cluster-based permutation test (Maris and Oostenveld, 2007). The cluster *p*-value was defined as the probability of observing a cluster of the larger mass over 10,000 random permutations. The mass of each cluster was calculated as the sum of signed t-scores in the adjacent vertices. The threshold for cluster inclusion was set to an uncorrected *p* < 0.025 for a two-tailed *t*-test. Positive and negative clusters were treated separately.

#### Sensor-space time-frequency data analysis

To analyze induced oscillatory activity, we extracted epochs that included a 1-s pre-stimulus and 2-s post-stimulus intervals locked to the presentation of the group ratings. The DC offset was removed from each epoch by aligning the time series to the average amplitude of a 1-s pre-stimulus interval. In order to remove the phase-locked activity, we subtracted the averaged evoked response from each epoch.

To estimate event-related changes in oscillatory power, we convoluted the signal with a family of 15 logarithmically spaced Morlet wavelets from 2 to 40 Hz. The mother wavelet had a time-resolution (FWHM) of 2 s at 1 Hz frequency. The event-related power perturbations (ERS/ERD) were indexed by computing the power ratios of 1-s post-stimulus to the 400-ms pre-stimulus baseline. We submitted the resulting ERS/ERD coefficients to a spatio-frequency permutation test with similar parameters as for the time domain data. The time and frequency information of the observed clusters was used for localization of the sources of the oscillatory activity.

#### Source space-time-frequency data analysis

To localize the sources of the oscillatory activity, we first band-passed the signal in theta (4–8 Hz) and beta-frequency bands (12–30 Hz). The band power was estimated as a standard deviation of the band-passed filtered signal in the 200–700 ms time window for the theta band and 500–1000 ms time-window for beta band, correspondingly. These exact shorter time windows were identified based on the visual inspection of the grand-averaged time-frequency maps. We then localized the sources of the power estimates for the theta band (for *conflict* trials) and beta band (for *no-conflict* trials) using the Brainstorm implementation of the MNE algorithm. Similarly, to the ERF analysis, we projected smoothed individual MNE solutions obtained for the aforementioned power components to obtain grand average source estimates.

## Results

### Behavioral results

Overall, in session 1 the participants rated the face as moderately trustworthy: mean rating = 4.3, SD = 0.67. During the second session participants changed their initial ratings toward the group rating in on average 45.8% of the trials SD = 6%. In 28% of trials, they kept their initial ratings without change, while in the remaining 26% of trials the subjects changed their rating in the opposite direction. A two-way ANOVA applied to the mean changes in ratings between sessions revealed a significant main effect of *conflict direction* [*F*_(1, 19)_ = 116.1, *p* = 0.00001] and a significant interaction for of *conflict direction* × *conflict size* [*F*_(1, 19)_ = 22.7, *p* = 0.00001]. *Post-hoc* Tukey HSD tests indicated significant differences between the mean rating changes in trials wherein the group opinion differed by ±3 points and trials wherein the group opinion and subjects' opinions matched (*p* < 0.01, Supplementary Figure 1). These results are summarized in Supplementary Table 1. We further analyzed the effect of social influence using a subset of faces with intermediate initial ratings (4 and 5) to account for possible artificial correlations caused by repeated measurements and the distribution of initial ratings. The two-way ANOVA also showed a significant main effect of *conflict direction*: *F*_(1, 19)_ = 12.54, *p* = 0.0007. Thus, similarly to previous findings, group opinion effectively modulated individuals' judgments of trustworthiness. This provides the conditions necessary for the following analysis examining brain correlates of exposure to group opinion.

### MEG results

#### Sensor space ERF analysis

**Figure 2:**
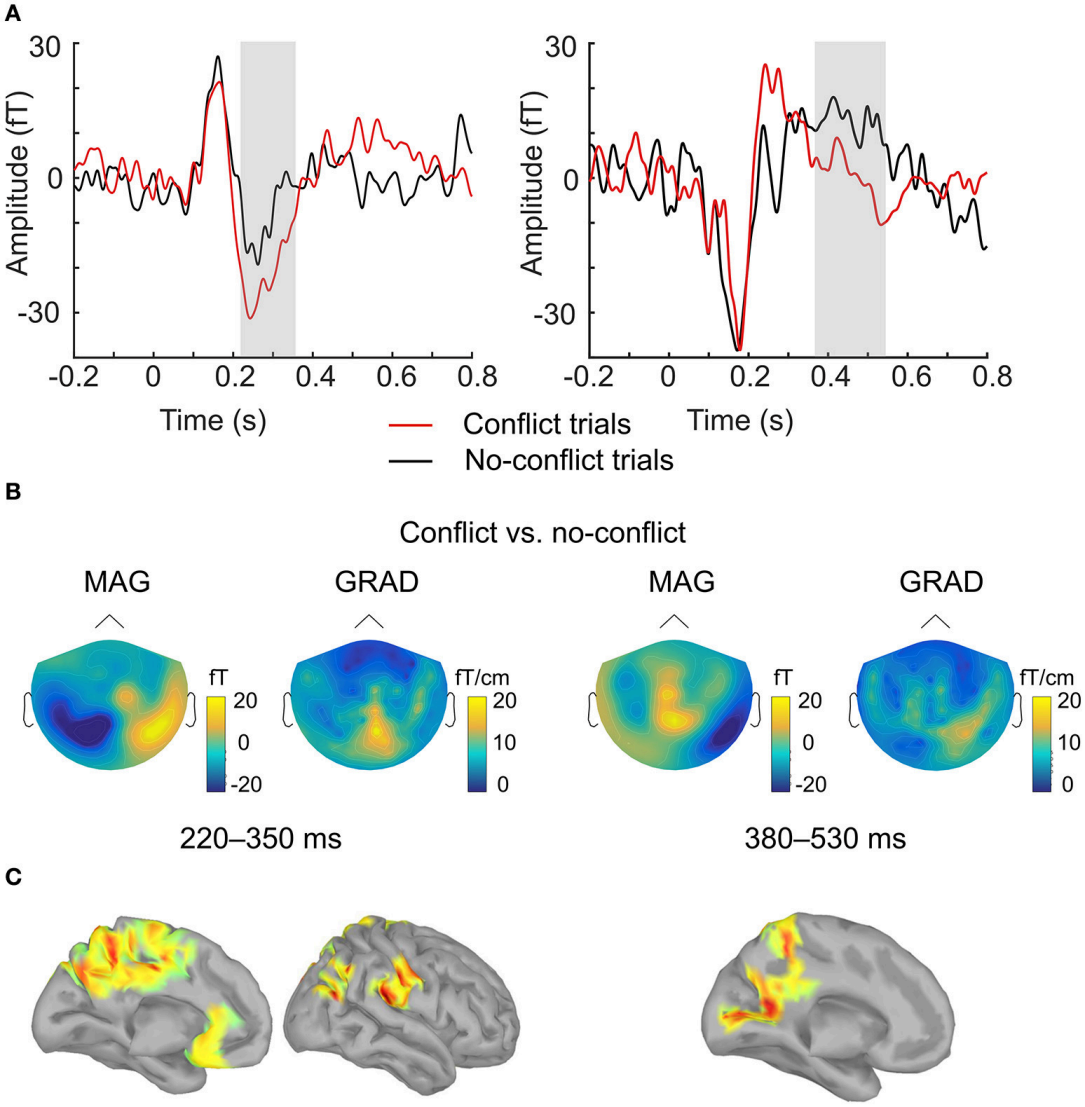
**Evoked response analysis. (A)** The grand-averaged event-related field in conflict (red) and no-conflict (black) trials. Time courses were obtained by averaging over magnetometers comprising two clusters identified by the permutation test. Gray boxes indicate the time-windows in which statistically significant differences were observed. **(B)** The grand-averaged difference in event-related field topographies (conflict–no-conflict) as measured by magnetometers (left) and the norm of planar gradiometer pairs (right) averaged over time-windows where statistically significant differences were observed on the sensor level. **(C)** Source reconstruction. Statistically significant clusters of sources displaying differences between conflict and no-conflict trials.

ERF analysis of magnetometer data identified two spatiotemporal clusters where the evoked activity in *conflict* trials differed significantly from the activity in *no-conflict* trials (Table 1). The first cluster occurred at 220–350 ms after the group ratings' onset, indicating a greater amplitude for *conflict* trials compared to *no-conflict* trials (Figure 2A, left, Figure 2B; left). The second cluster occurred at 380–530 ms, indicating a greater amplitude during *no-conflict* trials compared to *conflict* trials (Figure 2A, right; Figure 2B, right). Gradiometer data analysis revealed only one significant cluster of activity within 234–324 ms after group ratings' onset (Table 1). Interestingly, MEG evoked response amplitudes were largest for large (±3) conflicts with group opinion, intermediate for moderate (±2) conflicts and smallest for *no-conflict* trials (See Supplementary Figure 2).

**Table 1:** **Sensor space analysis: clusters of sensors showing significant differences between conflict and no-conflict trials**.

**ROI/condition**		**Sensor type**	**Time-window**	**Size**	**Sign**	***p*-value**
**TIME DOMAIN**						
		mag	224–348	1827	Negative	0.006
		mag	390–526	1615	Negative	0.012
		grad	234–324	756	Positive	0.004
	**Frequency, Hz**	**Sensor type**	**Time-window**	**Size**		***p*****-value**
**TIME-FREQUENCY DOMAIN**						
	2–5.9	mag	200–1000	153	Positive	0.048
	19.3–33.2	mag	200–1000	−101	Negative	0.048
	13.6–33.2	grad	200–1000	−315	Negative	0.016

#### Source-space ERF analysis of conflict-related activity

In agreement with our first hypothesis, group level permutation test on the source activation maps revealed statistically significant clusters showing greater activation in *conflict* as compared to *no-conflict* trials (Figure 2C; Table 2) in the following areas: the left and right posterior cingulate cortices (PCC including precuneus), the right temporal-parietal junction (TPJ), ventromedial prefrontal cortex (VMPFC), bilateral anterior cingulate cortices (ACC), and right superior occipital gyrus. No clusters showing significantly higher evoked responses for *no-conflict* trials as compared to *conflict* were observed.

**Table 2:** **Source space analysis: clusters of sources showing significant differences between conflict and no-conflict trials**.

**Analysis time-window**	**L/R**	**Structure**	**Cluster size**	**Cluster *p*-value, FWER**
220–350 ms	Right	PCC, Precuneus, SMA	1046	<0.001
	Left	PCC, Precuneus, SMA	812	<0.001
	Left	ACC, VMPFC	384	0.001
	Right	Occipital superior	150	0.013
	Right	ACC	129	0.018
	Right	DLPFC	68	0.059
380–530 ms	Left	PCC, Precuneus	213	0.038

#### Time-frequency analysis of conflict-related effects

In the time-frequency domain we observed two clusters where activity in *conflict* and *no-conflict* trials differed significantly. *Conflict* trials evoked greater increase in power of delta (2–3 Hz) and theta (4–8 Hz) activity in left posterior group of sensors. No-*conflict* trails evoked stronger increase in power in beta frequency range (13–30 Hz) over frontal-central group of sensors. Same analysis performed on gradiometers confirmed the beta-band cluster, whereas the lower frequency band cluster failed to reach statistical significance. The results of the sensor space time-frequency analysis are summarized in Table 1.

**Figure 3:**
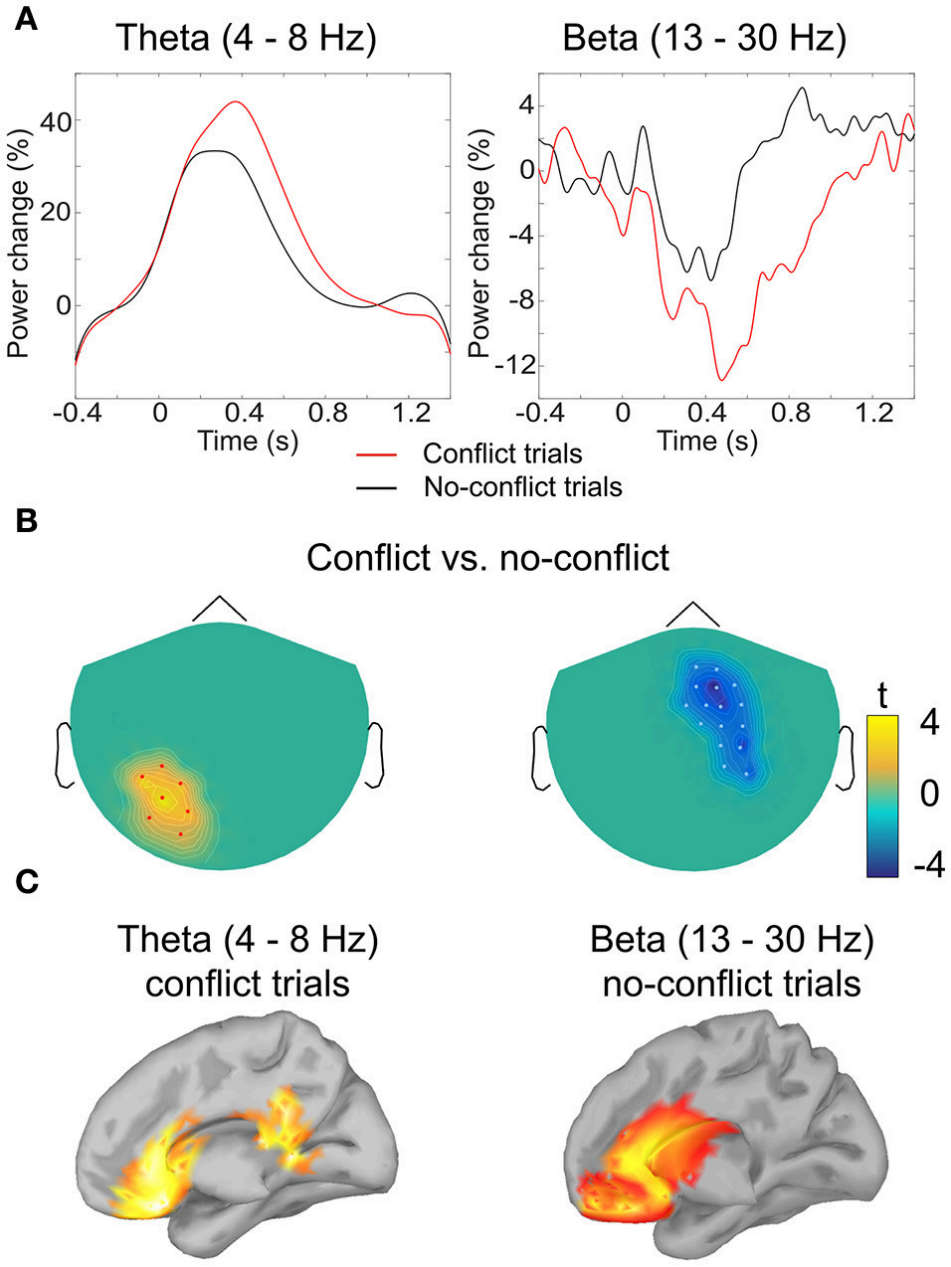
**Analysis of induced oscillatory activity. (A)** Grand-averaged event-related (de)synchronization of theta and beta band activity in conflict and no-conflict trials: amplitude envelope was averaged over the cluster of magnetometers comprising the cluster identified by the permutation test. **(B)** Clusters of sensors where event-related (de)synchronization in theta (left) and beta (right) band differed significantly between conflict and no-conflict trials. **(C)** Grand-averaged source localization of the band power components in theta and beta frequency bands.

In order to test the second hypothesis we conducted a *post-hoc* analysis of the activity in the theta band at the frontal sensors. Analysis revealed that in both *conflict* trials and *no-conflict* trials, the magnitude of frontal theta activity (4–8 Hz) increased relative to the pre-stimulus baseline (Figures 3A,B), as follows: mean magnitude increase = 17.3% (SD = 10.9) in the *conflict* trials; mean magnitude increase = 7.2% (SD = 6.7) in the *no*-*conflict* trials. The frontal theta activity was stronger in the *conflict* trials than in the *no*-*conflict* trials (mean magnitude difference = 10, SD = 7.7). This observation was supported by the one-way ANOVA performed for the theta ERS coefficients, *F*_(1, 19)_ = 7.79, *p* = 0.0088.

#### Source analysis of oscillatory activity

Source analysis of the power distributions in the theta band (4–8 Hz, 200–700 ms) indicated that the activity is produced by the ACC and PCC. For the beta band (13–30 Hz, 500–1000 ms) the main sources of oscillatory activity were localized at the ventromedial prefrontal cortex (VMPFC), and the rostral parts of the ACC (Figure 3).

## Discussion

The goal of this study was to clarify the temporal and spatial characteristics of electromagnetic brain responses to the social cues indicating either matching or mismatch between individual and group normative opinions. Initial sensor space analysis indicated that the perceived discrepancy with the group opinion modulated evoked magnetic fields in two time-windows: from 220 to 320 ms and from 380 to 530 ms after the presentation of the group opinion. The timing of the earlier evoked response corresponded to the time window of the FRN. Notably, these activations occurred within approximately the same time window as the conflict-related evoked response reported in a previous EEG study of social conformity (Shestakova et al., 2013). Source analysis revealed activations in the bilateral ACC, PCC including precuneus, left VMPFC and right TPJ as well as in the visual areas. Overall, our results suggest that perceived conflicts with a normative group opinion trigger medial cortical activity similar to the FRN or ERN—evoked responses, associated with performance monitoring and learning (Santesso et al., 2008; Walsh and Anderson, 2012; Luft, 2014).

Previous fMRI studies of social influence consistently reported an increase in the BOLD signal in the posterior MPFC following conflicts with group opinion (for a review, see Izuma, 2013). Contrary to these results, we did not observe significant evoked responses in the posterior MPFC. Instead, our results indicate more anterior (ACC) and posterior (PCC) medial sources of conflict-related activity, which is in line with reports on the localization of electromagnetic sources underlying FRN (Doñamayor et al., 2012a,b; Talmi et al., 2012). Moreover, research utilizing the multi-modal EEG-MEG-fMRI neuroimaging of closely-related error-related neural activity also revealed a more posterior source (PCC) for electromagnetic signatures of the ERN that was clearly distinct from the more anterior BOLD activation of the MPFC accompanying ERN (Agam et al., 2011). Thus, similar to other non-social studies, our results indicate the discrepancy between the hemodynamic and electrophysiological signals associated with processing negative outcomes. Overall, our MEG results (contrary to previous fMRI findings) suggest an important role of the PCC in monitoring the discrepancy of individual and group opinions. Further investigations are clearly needed to facilitate an understanding of the inconsistency of fMRI and MEG findings related to action-monitoring studies in general.

Our analysis of induced oscillatory activity provides further support toward performance-monitoring hypothesis of neural mechanisms of social influence. In trials, where individual opinion differed from the group's opinion, we observed an increase in power of low-frequency oscillations in theta band (4–8 Hz). These results are in line with the hypothesis suggesting that the FRN may reflect the perturbations of theta oscillations in the medial cortices (Cohen et al., 2008; Cavanagh et al., 2010). The source modeling of oscillatory activity recorded in our study indeed indicated that in the *conflict* trials theta activity occurred over multiple frontal regions including the ACC, VMPFC, PCC, partly overlapping with the sources of the evoked responses. As the increase in theta oscillatory power have been associated with unsigned prediction error signal (Cavanagh et al., 2012), the observed theta synchronization in *conflict* trials may indicate a strong expectation bias toward being in line with the group opinion.

In the *no-conflict* trials, where individual ratings matched the group rating, we observed the increase in power of beta oscillations occurring from 500 to 1000 ms post stimuli over the frontal-central sites. Previous neuroimaging studies robustly demonstrate an increase of oscillatory activity in beta (12–30 Hz) band following the delivery of rewards in gambling and learning tasks (Cohen and Ranganath, 2007; Marco-Pallarés et al., 2008, 2015; Doñamayor et al., 2011, 2012a,b). Marco-Pallarés et al. (2015) further hypothesized that beta band activity in the MPFC could underlie the coupling of fronto-striatal brain structures involved in learning from salient positive feedback. Our results suggest that being in line with normative group opinion may also activate the reward-processing neural circuitry, similarly to the non-social rewards (Izuma et al., 2008, 2010).

Most studies examining social influence primarily focus on error-related neural activity and post-error adaptation mechanisms, while whenever our opinion differs from social norms. Our results suggest that positive feedback mechanisms may also contribute to the effects of social influence. We show that being in line with the normative group opinion triggers stronger beta band oscillatory activity in the VMPFC, one of the key brain regions for processing reward information. These findings are in agreement with fMRI studies showing that socially rewarding events are associated with the activation of the VMPFC (for example, Rilling et al., 2002, 2004; Moll et al., 2006).

In line with the previous studies, we observed that subjects had a strong tendency to change their initial ratings toward the group opinion. However, we did not observe statistically significant differences in the evoked magnetic fields when comparing a subset of trials followed by changes in the initial rating toward the group rating and trials wherein the initial subjects' ratings were left unchanged. As MEG has a limited sensitivity for deeper cortical sources, such as ventral striatum and MPFC, the signal-to-noise ratio may not have been optimal for addressing this question.

Similar to previous studies using face judgment tasks, we used only female portraits and recruited only female subjects. This was done to avoid cross-gender ratings that could be related to mate selection and thus employ highly specific neural mechanisms (Cloutier et al., 2008) presumably less prone to social influence. Therefore, further studies are needed to generalize our findings to both genders.

Taken together, our results suggest that two generic learning mechanisms may underlie social influence. The first neural mechanism triggers a “reward prediction error”-like signal following the perceived opinion discrepancy. This mechanism activates the error-processing circuitry in the anterior and posterior medial cortices as indexed by the evoked activity and by the increase in power of frontal theta oscillations to prevent deviations from normative behavior (or group opinion). The second neural mechanism is underlined by activity of the VMPFC and ACC as indicated by an increase in power of beta oscillations. It may promote group coherence by reinforcing normative behavior, i.e., by rendering such behavior immediately rewarding. Overall, our results further contribute to the growing body of literature investigating the neural mechanisms of social influence, supporting the profound role of the medial cortices in neural mechanisms of social influence.

## Author contributions

IZ: Collected the MEG data, analyzed the data, wrote the manuscript; VK: Conceived and designed the experiment, wrote the manuscript; AO: Analyzed the MEG data, wrote the manuscript; VM: Analyzed the MEG data; AS: Designed the experiment, wrote the manuscript.

## Funding

The study has been funded by the Russian Academic Excellence Project “5–100.”

### Conflict of interest statement

The authors declare that the research was conducted in the absence of any commercial or financial relationships that could be construed as a potential conflict of interest.
