# Supplementary material for: MEG Signatures of a Perceived Match or Mismatch between Individual and Group Opinions
Source: Front Neurosci. 2017 Jan 23;11:10. doi: 10.3389/fnins.2017.00010 (PMC5253388; doi:10.3389/fnins.2017.00010)
Supplement: Supplementary file 1 [file Presentation1.PDF]

Supplementary Material

MEG Signatures of a perceived match or mismatch between individual and group opinions

Zubarev I*, Klucharev V, Ossadtchi A, Moiseeva V, and Shestakova A

Correspondence: Corresponding Author: ivan.zubarev@aalto.fi

1 Supplementary Figures and Tables

1.1 Supplementary Figures

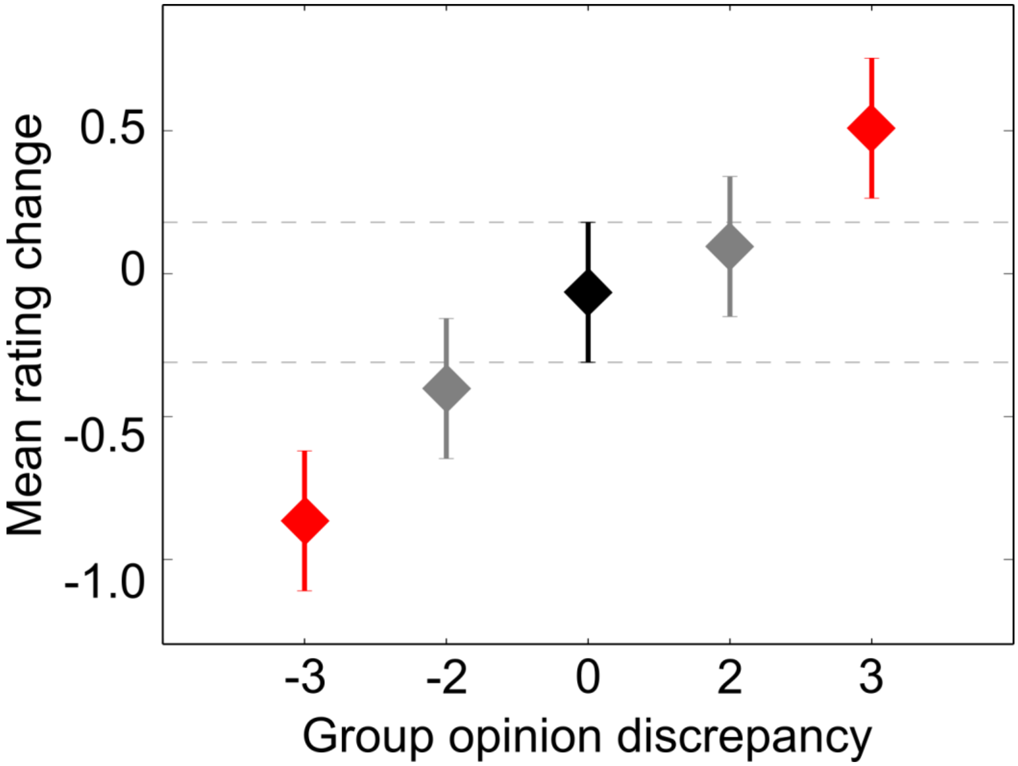

Supplementary Figure 1. Behavioral results: the mean face trustworthiness rating change between Session 1 and Session 2 in trials where the group rating was more negative (by 2 and 3 points), matched the subject's rating (0), and was more positive about the face (by 2 and three points). Bars indicate 95% confidence interval. Trial groups significantly different from 'match' condition ($p < 0.05$) are indicated in red.

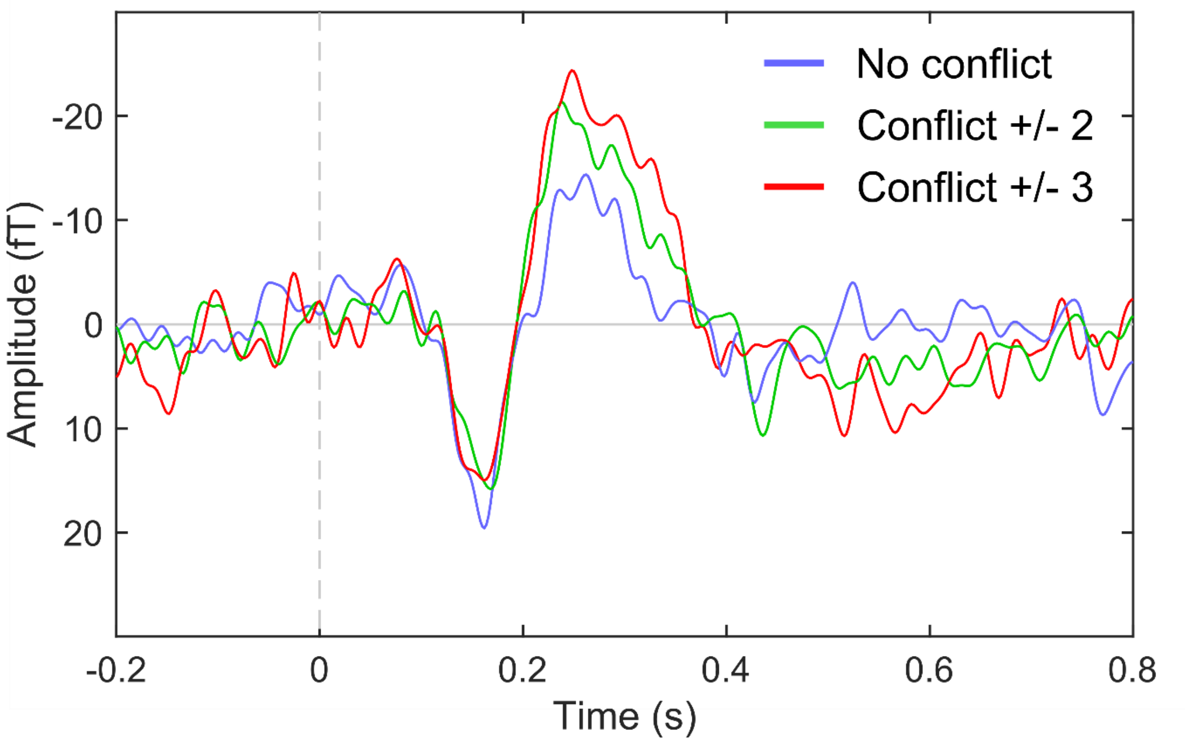

Supplementary Figure 2. MEG evoked responses for large (+/- 3) conflicts, moderate (+/-2) conflicts and no-conflict trials.

Supplementary Table 1. Post-hoc Tukey HSD tests on behavioral data

Trial group 1	Trial group 2	Mean diff	lower	upper	reject	p-value
-3	-2	0.4631	-0.0268	0.9529	False	0.218
-3	0	0.7997	0.3099	1.2896	True	0.001
-3	2	0.9599	0.4701	1.4497	True	0.001
-3	3	1.3739	0.884	1.8637	True	0.001
-2	0	0.3367	-0.1532	0.8265	False	0.638
-2	2	0.4968	0.007	0.9867	True	0.144
-2	3	0.9108	0.4209	1.4006	True	0.001
0	2	0.1602	-0.3297	0.65	False	0.9
0	3	0.5741	0.0843	1.064	True	0.047
2	3	0.414	-0.0759	0.9038	False	0.367
